# Antitumour effects of single or combined monoclonal antibodies directed against membrane antigens expressed by human B cells leukaemia

**DOI:** 10.1186/1476-4598-10-42

**Published:** 2011-04-19

**Authors:** Séverine Loisel, Pierre-Alain André, Josee Golay, Franz Buchegger, Jean Kadouche, Martine Cérutti, Luca Bologna, Marek Kosinski, David Viertl, Angelika Bischof Delaloye, Christian Berthou, Jean-Pierre Mach, Laurence Boumsell

**Affiliations:** 1 EA2216 and IFR148, University Medical School, Université Européenne de Bretagne, F-9238 Brest, France; 2 Service of Nuclear Medicine, University Hospital of Lausanne, CH-1011 Lausanne, Switzerland; 3 Laboratory of Cellular Therapy, USC Ematologia, c/o Presidio Matteo Rota, Ospedali Riuniti, I-24128, Bergamo, Italy; 4 Service of Nuclear Medicine, University Hospital of Geneva, CH-1211 Geneva 14, Switzerland; 5 MAT Biopharma, MAT Ltd., F-91030 Evry Cedex, France; 6 Centre National de La Recherche Scientifique (CNRS) UPS 3044, Unité Baculovirus et Thérapie F-30380 Saint-Christol-lès-Alès, France; 7 Institute of Applied Radiophysics, University of Lausanne, CH-1007 Lausanne, Switzerland; 8 Department of Biochemistry, University of Lausanne, CH1066 Epalinges, Switzerland; 9 Institut National de la Santé et de la Recherche Médicale, U1016, Université de Paris V, Paris, France

## Abstract

**Background:**

The increasing availability of different monoclonal antibodies (mAbs) opens the way to more specific biologic therapy of cancer patients. However, despite the significant success of therapy in breast and ovarian carcinomas with anti-HER2 mAbs as well as in non-Hodkin B cell lymphomas with anti-CD20 mAbs, certain B cell malignancies such as B chronic lymphocytic leukaemia (B-CLL) respond poorly to anti-CD20 mAb, due to the low surface expression of this molecule. Thus, new mAbs adapted to each types of tumour will help to develop personalised mAb treatment. To this aim, we analyse the biological and therapeutic properties of three mAbs directed against the CD5, CD71 or HLA-DR molecules highly expressed on B-CLL cells.

**Results:**

The three mAbs, after purification and radiolabelling demonstrated high and specific binding capacity to various human leukaemia target cells. Further *in vitro *analysis showed that mAb anti-CD5 induced neither growth inhibition nor apoptosis, mAb anti-CD71 induced proliferation inhibition with no early sign of cell death and mAb anti-HLA-DR induced specific cell aggregation, but without evidence of apoptosis. All three mAbs induced various degrees of ADCC by NK cells, as well as phagocytosis by macrophages. Only the anti-HLA-DR mAb induced complement mediated lysis. Coincubation of different pairs of mAbs did not significantly modify the in vitro results. In contrast with these discrete and heterogeneous *in vitro *effects, *in vivo *the three mAbs demonstrated marked anti-tumour efficacy and prolongation of mice survival in two models of SCID mice, grafted either intraperitoneally or intravenously with the CD5 transfected JOK1-5.3 cells. This cell line was derived from a human hairy cell leukaemia, a type of malignancy known to have very similar biological properties as the B-CLL, whose cells constitutively express CD5. Interestingly, the combined injection of anti-CD5 with anti-HLA-DR or with anti-CD71 led to longer mouse survival, as compared to single mAb injection, up to complete inhibition of tumour growth in 100% mice treated with both anti-HLA-DR and anti-CD5.

**Conclusions:**

Altogether these data suggest that the combined use of two mAbs, such as anti-HLA-DR and anti-CD5, may significantly enhance their therapeutic potential.

## Background

Monoclonal antibodies (mAb) have become an integral part in different treatments of lymphomas and leukaemias either as monotherapy or combined with chemotherapy and other antibodies. MAbs can be used in the form of unmodified antibodies or conjugated to radioactive elements or toxins. Anti-CD20 rituximab (Mabthera, Rituxan) has been extensively used and approved for the treatment of patients with various types of B-cell Non-Hodgkin Lymphoma (NHL). For the treatment of B-cell Chronic Lymphocytic Leukaemia (B-CLL), however, rituximab was found to be less successful at least in part due to lower expression of CD20. Thus, anti-CD52, alemtuzumab (Campath-1H) has been used and approved, but other mAbs are needed for the treatment of B-CLL. Many other mAbs directed against cell surface molecules of lymphoid leukaemic cells (CD4, CD19, CD22, CD23, CD30 CD40, CD74, CD80, HLA-DR, CCR4) or molecules over-expressed in tumour cells (CD71) are currently in clinical trial or in development. In this study, we will focus on three monoclonal antibodies directed against antigens strongly associated with the B-cell lymphoid leukaemia phenotype [1,2]: CD5, CD71 and HLA-DR.

CD5 is a marker of B-CLL, but is also expressed by the B1a subset of IgM secreting B cells and by most normal T cells. The biological role of this 65 kDa surface receptor is not clearly defined, but it seems to participate in immune tolerance as a negative regulator of B and T lymphocytes antigen receptor signalling and activation [3,4]. CD5 is overexpressed in B-CLL and represents one important parameter required for the diagnosis of B-CLL according to WHO criteria. It is also expressed by prolymphocytic leukaemia and mantle cell lymphoma, diseases with poor prognosis. The murine IgG_2a _CD5 antibodies T101 and anti-Leu-1, either unconjugated [5,6] or conjugated to toxins [7] or radioisotopes [8], have been tested in patients for therapeutic purpose. However, clinical benefits were limited or of short in duration. More recently, it was demonstrated that, *in vitro*, an anti-CD5 mAb can induce apoptosis of B cells from some patients with CLL and that cross-linking of CD5 on the surface of the cells was essential for apoptosis induction [9,10].

CD71 is the major transferrin receptor, a 95 kDa homodimeric (180kDa) type II transmembrane glycoprotein involved in the cellular uptake of iron and in the regulation of cell growth [11]. CD71 is widely expressed at low levels on normal cells and is overexpressed on cells with a high proliferation rate. Interestingly, different studies suggest that malignant tissues express CD71 at higher levels compared to their normal counterparts [12]. Moreover, CLL and NHL have increasing expression of transferrin receptor correlating with the clinical stage of the tumour [13]. Among several other mAbs, the murine monoclonal IgA antibody 42/6, directed against human CD71, demonstrated potent cytotoxic effects on haematopoietic tumour cells [14]. Following promising *in vitro *results, a phase I clinical trial was conducted on 27 patients with various refractory and advanced cancers of different origins [15]. The intravenous infusions treatment showed little side effects. Three patients, interestingly all of them with haematopoietic malignancies, demonstrated partial tumour response. The short duration of the remissions observed may be due to the rapid clearance of the IgA mAbs, suggesting that antibodies of different isotypes, such as chimeric and humanised mAbs could be more successful. More recently, a chimeric CD71 antibody called D2C has been reported to induce apoptosis and cell cycle arrest in G1 phase *in vitro *[16].

The human leukocyte antigen (HLA)-DR is a class II major histocompatibility complex (MHC) antigen which is a major player involved in the presentation of processed exogenous antigens to CD4+ helper T cells and thus in the initiation of the immune response. HLA-DR is expressed on several types of immune cells including B cells, activated T lymphocytes, monocytes and dendritic cells [17], and at a high level on B lymphoid leukaemias. Several studies have reported the ability of anti-HLA-DR antibodies to induce direct cell death (DCD) *in vitro *in either a caspase dependent or independent way [18,19]. However, our own *in vitro *studies suggest that DCD may be at least in part artefactual [20]. *In vivo*, the mechanism of tumour growth inhibition by anti-HLA-DR mAb has not been entirely clarified. Two anti-HLA-DR mAbs have been introduced in clinical trials in treatment of NHL: Lym-1 and Hu1D10. The radiolabelled murine IgG_2a _Lym-1 showed the first promising clinical results of radioimmunotherapy with increased survival of patients with either NHL or B-CLL [21]. Hu1D10 is a humanised antibody which, like Lym-1, was reported to bind to a variant of the HLA-DRβ chain. Hu1D10 has been evaluated in patients with relapsed or refractory indolent NHL where minimal toxicity and early responses have been observed [22]. However, phase II clinical trials were disappointing. Combination of Hu1D10 and rituximab has been recently evaluated with modest therapeutic results [23].

In order to investigate the potential therapeutic activity against CD5+ B malignancies of new murine monoclonal antibodies directed against CD5, CD71 and HLA-DR antigens, we evaluated their action, as single agents, and in combination of two mAbs. The target cell binding capacity, anti-proliferative, and apoptotic effects of the mAbs were evaluated *in vitro *on various lymphoma/leukaemia cell lines, or B-CLL cells from patients. *In vivo*, using two models of SCID mice i.p. or i.v. grafted with the human JOK1-5.3 cell line, we demonstrated the capacity of the mAbs to prolong the animal survival. Interestingly, a higher prolongation of the mice survival was observed when two mAbs, instead of a single one, were injected together.

## Results

### Binding of radio-iodinated mAbs to human leukaemia and lymphoma cells

Purified ^125^I-radiolabelled mAbs showed specific binding results of between 47 and 75% on cells expressing the target antigen, non-specific binding being below 1% on antigen negative cells (Table 1). Anti-CD5 mAb bound only to the T-cell line Jurkat and to the leukaemia cell line CD5+ JOK1-5.3, but not to the parental JOK1 cell line nor to the B lymphoma cell lines. Both anti-HLA-DR and rituximab, the latter taken as positive control, showed specific binding to all B-cell lines. The anti-CD71 mAb showed specific binding on all tested cell lines. Similarly, specific binding was detected by flow cytometry (data not shown).

**Table 1:** Percentage binding of ^125^I labelled purified mAbs to different leukaemia/lymphoma cell lines

mAbs	JOK1-5.3	JOK1	JURKAT	DAUDI	BL60.2
anti-CD5	61.6 (+/- 2.3)	< 1%	75.0 (+/- 1.8)	< 1%	< 1%
anti-CD71	47.2 (+/- 5.5)	n.d.	50.6 (+/- 6.5)	49.5 (+/- 2.6)	50.0 (+/- 2.6)
anti-HLA DR	56.1 (+/- 3.4)	50.7 (+/- 6.71)	< 1%	56.2 (+/- 13.0)	63.4 (+/- 20.1)
anti-CD20	41.0 (+/- 3.4)	40.8 (+/- 2.5)	> 1%	31.1 (+/- 5.6)	65.0 (+/- 6.2)

The percentage of binding of each ^125^I-labelled mAb to increasing numbers of each target cell lines was measured. Results are expressed as % of specific binding extrapolated to an infinite number of target cells [44]

### *In vitro *effects of native single mAbs or pairs of mAbs

**Figure 1:**
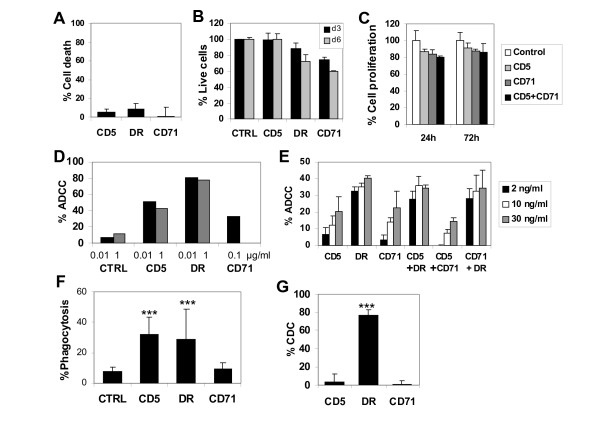
***In vitro *biological activity of monoclonal anti-CD5, anti-CD71 and anti-HLA-DR antibodies**. **A**, Induction of direct cell death or apoptosis measured after 24 hours treatment of JOK1-5.3 cells with 10 μg/ml mAbs, using the vital dye alamar blue. Results are % dead cells relative to control. CD5 = anti-CD5 mAb; DR = anti-HLA-DR mAb and CD71 = anti-CD71 mAb throughout. **B**, Effect on long term growth of JOK1-5.3, measured after 3 or 6 days treatment with 10 μg/ml antibodies and alamar blue. Data are % of live cells relative to untreated cells (CTRL) and are representative of at least 3 experiments. **C**, Percent proliferation of JOK1-5.3 cells, after incubation with single mAbs anti-CD5 or anti CD71 or the two mAbs combination, at a identical total concentration of 75 μg/ml, for 24 or 72 hours, as compared to untreated control cells, determined by incorporation of the radio-labelled thymidine analogue, ^125^I-Iododeoxyuridine. **D**, Induction of ADCC of JOK1-5.3 cells in presence of 0.01, 0.1 or 1 μg/ml antibodies as indicated, using purified NK cells at a 10:1 effector to target ratio. **E**, Comparison of ADCC of JOK1-5.3 cells in presence of single mAbs or pairs of anti-CD5+anti-HLA-DR, anti CD5+anti-CD71, or anti-CD71+anti-HLA-DR, at the total concentrations of 2, 10 or 30 ng/ml, using NK cells at a 10:1 effector to target ratio. **F**, Induction of phagocytosis of B-CLL targets by *in vitro *differentiated macrophages with 0.1 μg/ml antibodies. All data are representative of at least 3 experiments. **G**, Complement dependent cell lysis of JOK1-5.3 cells with 10 μg/ml antibodies and 20% human serum.

In order to determine the biological activities of the different mAbs, their potential effects on cell death after 24 hour incubation and on cell growth after 3-6 days was evaluated using the vital dye alamar blue. MAb anti-CD5 did not induce significant direct cell death of JOK1-5.3, Daudi or Jurkat cell lines, at concentrations up to 500 nM (75 μ g/ml) at 24 hours (Figure 1A and data not shown), nor did the antibody induce proliferation inhibition at day 3-6 (Figure 1B). MAb anti-CD71 did not induce cell death at 24 hours but induced 25-60% inhibition of JOK1 5.3 cell growth, observed after 3-6 days of culture (Figure 1B). Even more rapid and effective inhibition of Daudi and Jurkat cell growth was observed with anti-CD71 already at 48 hours using a ^125^I-IdUrd incorporation assay (60 and 25% of initial values, respectively, data not shown), confirming that anti-CD71 BA120 mAb can inhibit cell proliferation.

MAb anti-HLA-DR did not induce significant cell death at 24 hours, as measured by alamar blue vital dye (Figure 1A). However the antibody induced strong aggregation of JOK1-5.3, Daudi and Raji, due to homotypic adhesion as previously described [20,24,25]. Interestingly, this type of aggregation could lead to the artefactual appearance of direct cell death, as determined by flow cytometry analysis of a minority of cells after the exclusion of aggregated live cells [20]. Indeed, examination of cytospin preparations of mAb treated and 7AAD stained cells revealed only 2-5% of cell death after anti-HLA-DR treatment, confirming the alamar blue results (data not shown and [20]). The absence of apoptosis induction by our anti HLA-DR mAb on Daudi cells was confirmed by negative DNA laddering assay (data not shown). Interestingly, the B-cell line, BL60.2, which has a slightly lower surface expression of HLA-DR than Daudi cells (0.25 × 10^6^, molecules per cells, as compared to 10^6^, respectively) did not form any aggregates and showed no evidence of apoptosis by annexin V flow cytometry analysis after anti-HLA-DR mAb treatment. In long term culture of JOK1-5.3 (3-6 days), the anti-HLA-DR mAb induced only limited growth inhibition (11 and 18% respectively) (Figure 1B and data not shown). No growth inhibition of Daudi and Jurkat cell lines at 48 hours was observed by ^125^I-IdUrd incorporation assay with this anti-HLA-DR antibody (data not shown).

Thus, except for the known proliferation inhibition of the anti-transferrin receptor (CD71) mAb, the 3 mAbs analysed did not directly induce any significant cell death or inhibition of proliferation *in vitro *(Figure 1A and 5B).

In addition, coincubation of the target cell line JOK1-5.3 *in vitro *with different pairs of the 3 native mAbs did not induce detectable additional biological effects on the target cells. For instance, addition of the anti-CD5 mAb to anti-CD71 mAb did not induce any significant increase of proliferation inhibition of the JOK1-5.3 cells, as determined by ^125^I-IdUrd (Figure 1C). Similarly, the addition of anti-CD5 or anti-CD71 to anti-HLA-DR mAb did not modify the anti-HLA-DR mediated aggregation of JOK1-5.3 cells nor did it produce any new apoptosis induction properties (data not shown).

### Antibody dependent cellular cytotoxicity (ADCC)

ADCC is thought to be one of the most important mechanisms of action of therapeutic antibodies. In order to evaluate whether our anti-CD71, anti-HLA-DR and anti-CD5 mAbs were active in the mediation of ADCC by NK cells, CFSE labelled JOK1-5.3 were used as targets, after precoating with the different mAbs, followed by incubation with purified NK cells during 4 hours. Target cell death was next determined by 7AAD staining among CSFE labelled target cells by FACS analysis. As shown in Figure 1D, all mAbs mediated significant ADCC, with highest activity obtained by the anti-HLA-DR mAb (78-88% lysis), followed by anti-CD5 (43-55%) and anti-CD71 (33%) compared to 7-11% for control. ADCC experiments performed were always including controls with NK cells and target cells alone, or target cells preincubated with mAbs, which showed less than 5% dead cells and therefore the specificity of the ADCC reaction.

In separate experiments, using different donors of NK cells, the induction of ADCC by coincubation with different pairs of mAbs, such as anti-CD5 and anti-HLA-DR, anti-CD5 and anti CD71, or anti-CD71 and anti-HLA-DR, was compared with the incubation of the different single mAbs at 3 identical total concentrations of the single mAbs and the pair of mAbs. As shown in Figure 1E, the pairs of mAbs directed against different surface markers did not induce additive or synergistic ADCC effect.

### Phagocytosis of mAb-opsonised B-CLL cells by human macrophages

Phagocytosis by tissue macrophages is also a likely important mechanism of action of therapeutic mAbs *in vivo *[26]. Phagocytosis assays were performed by coincubation of *in vitro *differentiated macrophages with B-CLL targets in presence or absence of different mAbs, followed by microscopic evaluation of stained phagocytic cells [27]. The results of repeated experiments with the different mAbs compared to rituximab, as positive control, show that both anti-CD5 and anti-HLA-DR antibodies, but not anti-CD71, could mediate phagocytosis of target cells by macrophages. Indeed a mean of 29-33% phagocytosis was observed with these two mAbs (Figure 1E), compared to 37% with rituximab (data not shown). In contrast anti-CD71 did not induce significant phagocytosis compared to the negative control (Figure 1E).

### MAb induced complement-mediated lysis

Complement-mediated lysis (CDC) may play a role in the mechanism of action of mAbs [28]. Since the induction of complement activation is dependent on both Ig isotype and density and structure of the antigen recognised by the mAb on the target cells surface, we tested the capacity of our 3 mAbs to activate human complement. As shown in Figure 1C, the anti-HLA-DR mAb induced a strong lysis of almost 80% of JOK1-5.3 cells, while no significant lysis of the same cell line was observed with either anti-CD5 or anti-CD71, even though this cell line was positive for all 3 antigens.

### *In vivo *anti-tumour efficacy of mAbs in SCID mice

To determine the *in vivo *antitumour activity of the different mAbs, we established two xenograft models of the JOK1-5.3 cell line in mice, injected either i.p. or i.v.

**Figure 2:**
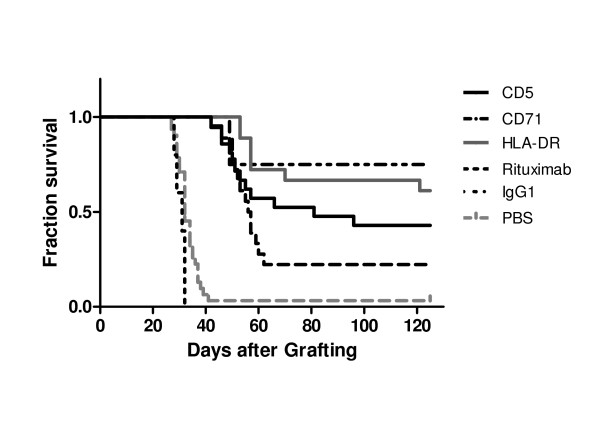
**Antitumour efficacy of the single mAbs in the intraperitoneal JOK1-5.3 model**. Kaplan-Meier curves showing survival fractions of SCID mice intraperitoneally grafted with JOK1-5.3 cells and treated with 2 i.p. injections at day 1 and 4 of 0.5 mg of mAbs anti-CD5, Anti-CD71, anti-HLA-DR and rituximab. Controls were either untreated mice (PBS) or mice treated with an irrelevant mouse IgG_1 _isotype. (All treated groups were significantly different from the control groups p < 0.005). The study was performed twice. Data are presented as cumulative percentage of both experiments.

In the first intraperitoneal model, therapy studies were performed in SCID mice with i.p. grafted JOK1-5.3 leukaemia. All mice treated with 2 i.p. injections of 0.5 mg of mAb anti-CD5, CD20, CD71 or anti-HLA-DR, at 24 and 96 h after grafting, showed highly significant (p < 0.005) improvement of survival (Figure 2 and Table 2). Median survival time was extended, from about 32 days in controls, to 56 days after anti-CD71 treatment and was not reached after the anti-HLA-DR treatment during the four-month observation period. At study end, absence of any sign of tumour at study end was observed in 4 of 18 (22%) mice treated with anti-CD71 and in 11 of 18 mice (61%) treated with anti-HLA-DR mAb. The anti-CD5 treatment resulted in 4 months survival without any sign of tumour in 9 of 21 mice (43% mice), while tumour growth delay of about 14 days was observed in the 12 other mice. Rituximab, injected as a positive control in the same treatment regimen of the JOK1-5.3 i.p. tumour model, led to a significant survival advantage with complete response observed in 3 of 4 treated mice (Figure 2).

**Table 2:** Comparison of antitumour efficacy of single and combined mAbs in the intraperitoneal tumour model

Injected mAbs	Medium survival in days	N° of tumour free animal at day 120 (%)	
**Anti-HLA-DR**	> 120*	11/18	(61%)
**Anti-CD5**	81*	9/21	(43%)
**Anti-CD71**	56*	4/18	(22%)

**Anti-HLA-DR + anti-CD5**	> 120**	13/13	(100%)
**Anti-CD71 +anti-CD5**	> 120**	7/8	(88%)
**Anti-CD71 + anti-HLA-DR**	> 120	7/13	(54%)

**Untreated animals**	32	1/47	(2%)

*significantly different from irrelevant IgG injected control mice (p < 0.005)

**significantly different from both single antibody treatments (p < 0.05)

Ascites development was generally observed in untreated mice at the same time as in mice treated with control IgG_1_, requiring their sacrifice. In contrast, ascites formation was observed only in a small minority of antibody treated mice (3.4%, 2/57 mice). However, in many treated mice the tumours developed as s.c. inguinal and/or intraperitoneal nodules (data not shown). The mice were sacrificed when tumour load reached a volume of 1 cm3, and scored as dead. Injection of an irrelevant, isotype matched control murine IgG_1 _had no significant effect, as compared with PBS injected mice.

For the anti-CD5 murine IgG_2a _antibody, additional control experiments were performed with mice bearing the wild type JOK1 tumour that does not express measurable amounts of CD5. This cell line was shown in separate experiments to produce a median survival of 32 days when grafted i.p. in SCID mice, similar to JOK1-5.3. As expected, treatment with the anti-CD5 mAb had no effect on JOK1 wild type tumours.

**Figure 3:**
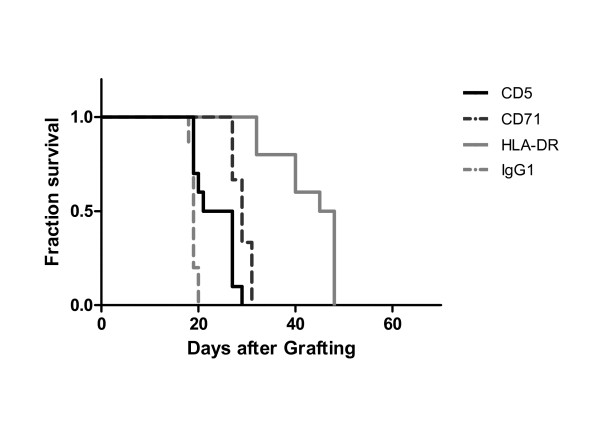
**Antitumour efficacy of the single mAbs in the intravenous JOK1-5.3 model**. Kaplan-Meier curves showing survival fractions of SCID mice grafted by i.v. injection via the tail vein of 10 × 10^6 ^JOK1-5.3 cells and treated with i.v. injections of either mAb anti-CD5, anti-CD71 or anti-HLA-DR (0.25 mg on days +3, +5, +7 and +11) or irrelevant mouse IgG_1 _isotype. All treated groups are significantly different from the control groups p < 0.005). The study was performed twice. Data are presented as cumulative percentage of both experiments.

In the second intravenous model, JOK1-5.3 cells were inoculated i.v. into SCID mice. Mice received four i.v. injections of 0.25 mg from the same murine anti-CD5, CD71 and anti-HLA-DR mAbs or irrelevant control IgG. All control mice had to be sacrificed because of hind limb paralysis with median survival times of 18.5 days. Mice that received mAb treatment had a significant (p < 0.05) or highly significant (p < 0.005) prolongation of survival when compared to mice that received control antibody (Figure 3). Median survival time was extended from 18.5 days in controls to 25 days, 29 days and 45 days after anti-CD5, anti-CD71 and anti-HLA-DR treatment, respectively. There was no statistically significant difference between the survival curves using anti-CD5 or anti-CD71. Despite survival prolongation, all mAbs-treated mice eventually developed hind limb paralysis.

### Treatment with different combinations of two mAbs improves the *in vivo *response

**Figure 4:**
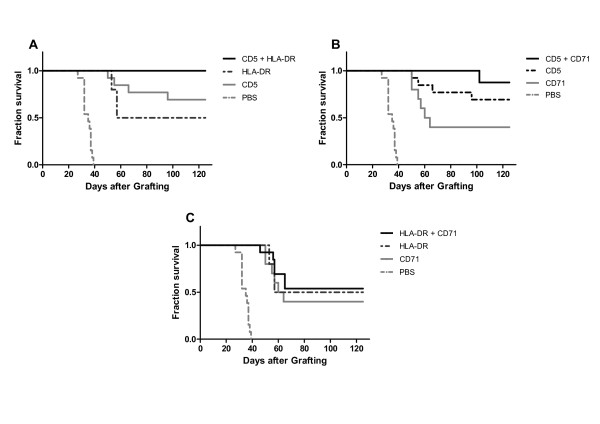
**Increased antitumour efficacy of the injection of two mAbs combination in the intraperitoneal JOK1-5.3 model**. Kaplan-Meier curves showing survival fractions of SCID mice intraperitoneally grafted with JOK1-5.3 and treated with two i.p. injections of different combinations of two mAbs (0.25 mg of each mAb) at day 1 and 4: **A**, combination of anti-HLA-DR and anti-CD5, **B**, combination of anti-CD71 and anti-CD5 or **C**, combination of anti-HLA-DR and anti-CD71 mAbs. Combination treatments were compared with two i.p. injections of 0.5 mg from only one mAb at day 1 and 4, or PBS injected control mice. The study was performed twice. Data are presented as cumulative percentage of both experiments.

We next investigated whether combined treatment with 2 different antibodies could improve therapeutic response in the same i.p. and i.v. tumour models. In the i.p. model, the combined treatment with anti-HLA-DR and anti-CD5 (Figure 4A and Table 2) led to a complete response in 13 of 13 animals (100%), none of the mice showing any sign of disease after 4 months. The combination of anti-CD5 and anti-CD71 also showed very favourable results, with only 1 mouse out of 8 developing a subcutaneous tumour, while 7 animals (88%) were apparently cured (Figure 4B and Table 2). The results from both of these treatment combinations, anti-HLA-DR/anti-CD5 and anti-CD5/anti-CD71 yielded a significant increase in survival compared with treatment by the individual mAbs alone, using the same quantity as the total of the combined mAbs injections. The combination of anti-HLA-DR and CD71 mAb resulted in tumour growth inhibition and apparent cure in 7 of 13 mice (54%). However, this result was not significantly different from that obtained with the anti-HLA-DR mAb used alone (Figure 4C).

**Figure 5:**
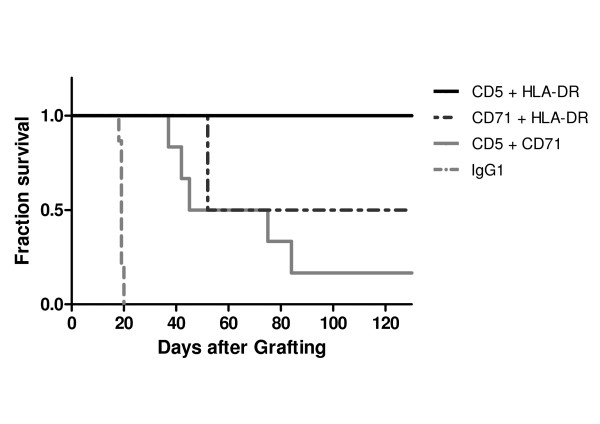
**Increased antitumour efficacy of the injection of two mAbs combination in the intravenous JOK1-5.3 model**. Kaplan-Meier curves showing survival fractions of SCID mice intravenously grafted with JOK1-5.3 and treated by i.v. injections of different combinations of two mAbs (0.25 mg on day 3, 5, 7 and 11): combination of anti-HLA-DR plus anti-CD5, anti-HLA-DR plus anti-CD71, or anti-CD71 plus anti-CD5 mAbs. Controls were mice injected with PBS. The study was performed twice. Data are presented as cumulative percentage of both experiments.

In the i.v. model of JOK1-5.3 bearing SCID mice, treatment with the three possible combinations of murine antibodies resulted in prolongation of mice survival, as compared with placebo-treated controls (Figure 5). The combination of anti-CD5 and anti-HLA-DR confirmed the high efficacy of this combination already observed in the i.p. model, with complete response observed in all animals (12/12, 100%) and none of the mice showing any sign of disease after 10 months (Figure 5). As shown in Table 3, the median survival times of animals treated with anti-HLA-DR plus anti-CD5 mAbs was more than 300 days, representing an apparent cure, while treatment with anti-CD71 plus anti-HLA-DR mAbs led to a 52 days median survival (52-53) and 6/12 mice (50%) cured. Treatment with anti-CD71 plus anti-CD5 mAbs gave a slightly lower median survival of 45 days (37-82) and only 2/12 (17%) of mice cured, but this has to be compared with a median survival of only 18.5 days (19-20) and no cure for PBS treated mice.

**Table 3:** Comparison of antitumour efficacy of single and combined mAbs in the intravenous tumour model

Injected mAbs	Median survival in days	N° of tumour free animal at day 300 (%)	
**Anti-HLA-DR**	45*	0/10	(0%)
**Anti-CD5**	25*	0/10	(0%)
**Anti-CD71**	29*	0/6	(0%)

**Anti-HLA-DR + anti-CD5**	> 300**	12/12	(100%)
**Anti-CD71 + anti-CD5**	45**	2/12	(17%)
**Anti-CD71 + anti-HLA-DR**	52**	6/12	(50%)

**Untreated animals**	18.5	0/36	(0%)

*significantly different from irrelevant IgG injected control mice (p < 0.005)

**significantly different from both single antibody treatments (p < 0.05)

## Discussion

This report was aimed at analysing the activities of three new mAbs, directed against different cell surface molecules preferentially expressed by B-CLL and other haematopoietic tumours: CD5, the transferrin receptor or a class II MHC molecule. We started our evaluation of the mAbs by an *in vitro *analysis of their biological effect, without or with effector cells, on different target leukaemia/lymphoma cell lines, expressing the relevant molecules. Except for some proliferation inhibition by anti-CD71, none of the antibodies showed significant direct cell death induction at 24-48 hours. In contrast, all antibodies showed immune mediated activities, such as CDC (anti-HLA-DR), phagocytosis (anti-CD5 and anti-HLA-DR) and ADCC (all 3 mAbs with different efficiencies). Interestingly, *in vitro*, the incubation of target cells with different pairs of mAbs did not induce any significant superior anti-tumour effect.

We then tested the MAbs in a first *in vivo *model, consisting of the direct injection of both the tumour cells and the antibodies, after 24 hours, in the peritoneal cavity of immunodeficient SCID mice. Encouraged by the significant specific anti-tumour effect of the three mAbs, we moved to a more relevant tumour model with target cells injected in the tail vein of the same type of mice, followed by the systemic injections of the mAbs by the same route. The systemic injection of the mAbs confirmed and extended the encouraging anti-tumour effects obtained in the peritoneal cavity model. Then, we moved to the second part of our experimental therapy analysis, which demonstrates that the coinjection of different pairs of two mAbs in the same animals can give significant improvements of anti-tumour effect as compared to single mAb injection.

The fact that our three mAbs had efficient anti-tumour effect *in vivo*, in both our local i.p. and systemic i.v. models, as well as mostly immune mediated effects *in vitro*, strongly suggests that our antibodies are acting *in vivo *with the help of autologous biological mediators, such as effector cells and/or complement. In addition a direct antiproliferative effect of anti-CD71 may play a role. However, it remains difficult to determine which of the different effector mechanisms for each mAb is playing the most important role in the induction of the observed anti-tumour effect. In this context, it is worth mentioning that even for the most successful anti-cancer therapeutic mAbs broadly used in the clinic, such as anti-CD20, anti-HER2 or anti-EGF receptor, the exact mechanism of anti-tumour activity has not yet been entirely elucidated. For anti-EGF receptor mAbs, it is claimed that they block the accessibility to the different EGFR ligands, but for anti-HER2, which is an orphan receptor without ligand, this type of mechanism does not apply.

A few years ago, there was almost a consensus that ADCC by NK cells and phagocytosis by macrophages were indeed the most important mechanisms of mAb cancer therapy. This opinion was initially based on experimental results showing that in nude mice knocked-out for Fcγ receptor, the anti-HER2 and anti-CD20 mAbs were inefficient to treat human tumours expressing the relevant target antigens [29], and second, on the clinical observations of a correlation between FcγR3A genetic polymorphisms and response to mAb therapy [30,31].

However, recent experimental results showing that the F(ab')_2 _fragments of anti-EGFR and anti-HER2 mAbs could significantly inhibit the growth of pancreatic carcinoma xenografts in nude mice, clearly demonstrate that at least part of the anti-tumour effect is not ADCC mediated and must be due to binding of the F(ab')_2 _fragments to the sensitive HER1 and HER2 receptors [32]. Furthermore, the fact that the most successful mAbs, clinically accepted for cancer therapy, are directed against sensitive surface receptors (EGFR and HER2) or calcium pump like structure (CD20), suggests that at least part of the anti-tumour effect of mAbs is induced by their capacity to activate or block a pathway of internal signalling, leading to inhibition of tumour cell proliferation. In relation to our tumour model system, it is interesting to note that the CD5 molecule was previously shown to act as negative regulator of T or B cells [33]. However, the absence of additive effect of the coincubation of two mAbs on target cells *in vitro*, is not in favour of the hypothesis that the superiority of our two mAbs *in vivo *treatment would be due to a cooperative inhibitory pathway.

Concerning the role of complement, its activation at the tumour site should help control tumour through C3b opsonisation of tumour cells, or by the release of C3a or C5a anaphylatoxins, which should attract immune cells, increase the vasodilatation and permeability of tumour vessels and thus the accessibility of more antibody molecules. However, those who think that tumour growth is entirely dependent on vascularisation [34] may be against the induction of complement activation. Similarly, the activation of complement by anti-CD20 has been demonstrated, but the possible positive or negative role of complement in tumour therapy is still the subject of debate [28].

Regarding the question of *in vitro *cytotoxicity of anti-HLA-DR antibody, in the course of the *in vitro *study, we made the following original observation. Anti-HLA-DR mAbs were known to induce cell aggregation, through homotypic adhesion, of cell lines expressing this antigen and were reported, on the basis of flow cytometry analysis after annexin V, or propidium iodide staining, to induce up to 80% cell death [35]. However, after reproducing these results with our own anti-HLA-DR mAb on Daudi and JOK1-5.3 cells, we found that these high cytotoxicity results were likely to be artefactual due to the exclusion in the analysis of live aggregated cells, resulting in the detection of apoptosis on a minority of cells remaining in single cell suspension. In contrast, when the bulk of antibody treated cells were tested without preselection on cytospin preparations, by 7AAD staining, we found only a minority of dead cells [20]. Interestingly the B-cell line, BL60.2, which was found to express 4 times less HLA-DR molecules than Daudi cells and does not present any aggregation upon treatment with anti-HLA-DR mAb did not show either any evidence of mAb induced cytoxicity, when tested by annexin V staining and conventional flow cytometry analysis (PAA unpublished observations). This represents an additional argument in favour of our interpretation that the flow cytometry evidences of anti-HLA-DR induction of direct cytotoxicity are artefactual.

The simultaneous attack on tumour cells by the coinjection of two mAbs represents a promising new therapeutic strategy, whose mechanism of action is far from being understood. Among the few examples in the literature, the coinjection of anti-CD20 and anti-CD22 mAbs has already been clinically evaluated in the treatment of non-Hodgkin lymphomas. However, despite the description of some durable complete tumour responses, the advantage of adding the humanised anti-CD22, epratuzumab to the standard treatment with anti-CD20 rituximab, has not yet been convincingly demonstrated [36]. Similarly, several early clinical trials testing the coinjection of alemtuzumab with rituximab were reported with good tolerance, but not yet the demonstration of a clear clinical benefit [37,38].

Experimentally, two other coinjections of mAbs have been reported to induce synergistic anti-tumour effect. The first associated the injection of two mAbs directed against EGF and VEGF receptors, which were not expressed by the same target cells. Thus, the synergistic effect was attributed to a double action of the anti-EGFR directly on tumour cells and of the anti-VEGFR on the tumour vascular component [39]. The second consisted in the coinjection of mAbs directed against two receptors of the HER family, matuzumab anti-EGFR (HER1) and trastuzumab, anti HER2. These two mAbs showed a clear synergistic therapeutic activity, as compared to single mAb injection, against two human pancreatic carcinoma xenografts, expressing the two receptors [40]. Here again, the mechanism of the synergistic effect was not fully understood, but was likely due to inhibition of the dimerisation of the two receptors, which is known to represent a central activation signal. Furthermore, in this case, the anti-tumour synergism was not due only to an increase of NK cells recruitment, since the anti tumour synergism was confirmed by the use of F(ab')_2 _fragments from the two mAbs [32].

## Conclusions

We have demonstrated here the higher anti-tumour effect of the combined injection of two mAbs, as compared to single mAb injection. The fact that the combined mAbs anti-tumour effect was not observed in vitro, but only *in vivo*, strongly suggest that the therapeutic advantage obtained with different pairs of mAbs is due to multiple antibody induced effector mechanisms, such as ADCC, phagocytosis and/or CDC. In some mAbs combination, we cannot rule out a simple additive effect due to the involvement of two mAbs reacting with independent antigens expressed by the tumour cells, but in the case of the coinjection of anti-HLA-DR and anti-CD5, or anti-CD71 and anti-CD5 mAbs, the clear advantage of same amounts of mixed mAbs over single mAb, as demonstrated in the i.p. model, are definitely in favour of a synergistic anti-tumour effect. This synergism associated with different mechanisms of action of the antibodies, is likely to be responsible for the complete inhibition of tumour growth after i.v. coinjection of anti-HLA-DR and anti-CD5 mAbs and suggest that the systemic injection of such pairs of mAbs represents a promising therapy strategy. Mouse-human chimerisation or humanisation of the mAbs, as well as synthesis of bispecific antibodies, with binding sites selected from the best pairs of mAbs, will have a good chance to further improve the observed therapeutic efficiency.

## Methods

### Cells and Reagents

The human Hairy Cell Leukaemia (HCL) JOK1 (CD5-, CD71+, HLA-DR+, CD20+) was stably transfected with pLNCX vector carrying human CD5 cDNA. Briefly, sublclone JOK1-5.3 stably expressing human CD5+ on the cell surface was selected by limiting dilution and expanded *in vitro*. The cells stably maintained CD5 expression, as determined by standard immunophenotyping and FACS analysis (MFI22) to levels similar to that observed in B-CLL for up to 6 months continuous culture ([41] and Le Ster K et al., manuscript in preparation). We verified that JOK1-5.3 had the following phenotype: CD5+, CD71+ HLA-DR+ and CD20+. The Human Burkitt's lymphoma cell lines Daudi (American Type Culture Collection, ATCC) and BL60.2 [42], (kindly provided by Dr Stephan Mathas, Max-Delbrück-Center for Molecular Medicine, Berlin, Germany) are CD5-, CD71+, HLA-DR+, CD20+, while the Human Jurkat T-cell leukaemia line (ATCC) is CD5+, CD71+, CD20- and HLA-DR-. All cells were grown in RPMI1640 medium (Gibco, Invitrogen) supplemented with 10% fetal calf serum, 2 mM l-glutamine, 150 μg/ml penicillin-streptomycin (Gibco, Paisley, Scotland).

Heparinised peripheral blood was obtained after informed consent from patients with B-CLL at diagnosis. All patients were diagnosed by routine immunophenotypic, morphologic, and clinical criteria. In all cases, double staining with CD19 and sIg was performed, allowing us to establish monoclonality and to determine the percentage of neoplastic versus normal B cells present in the sample. The cells were separated on a Ficoll Hypaque gradient (Seromed, Berlin, Germany).

The murine monoclonal antibodies anti-CD5 (O490, IgG_2a_), anti-CD71 (BA120G, IgG_1_) and anti-HLA-DR (BK267W, IgG_1_) were developed by the laboratory of L. Boumsell (INSERM U567, France) and kindly produced and purified by the MAT Biopharma laboratory (MAT Biopharma Evry, France). BK267 was shown to recognise HLA-DR molecules by tissue distribution and Western blot. A mouse anti-CEA mAb of IgG_1 _subclass was used as control immunoglobulin for IgG_1 _antibodies. The chimeric anti-CD20 monoclonal antibody rituximab and the humanised anti-CD52 antibody alemtuzumab (Campath-1H) were obtained from Roche Pharma, Basel, Switzerland and Bayer Schering Pharma, Berlin, Germany respectively.

### Radiolabelling of mAbs and binding measurements

The radiolabelling of mAbs with ^125^I was performed by the Chloramine T method using 37MBq/mg of protein (1mCi/mg) [43] and purified on Dowex ion exchange column. The radiochemical purity of the different labellings was controlled by Instant Thin Layer Chromatography using 85% MeOH buffer as mobile phase and/or by HPLC using size exclusion column BIOSep SEC S3000.

To assess the antigen binding specificity of the monoclonal antibodies to the different cell lines, fixed amounts of ^125^I-labeled-mAb (6ng~7500 cpm) were incubated with various numbers of cells (2-10 × 10^6 ^cells) in 100 μl of PBS (Phosphate Buffer Saline 0.16M, pH7.4), 2% FCS for 2 hours at 4°C. Non-specific binding was determined by specific binding inhibition with an excess (100 μg) of unlabelled antibody. Cells were then washed with ice-cold PBS/FCS to remove unbound mAbs and cell-bound activity was measured on a γ-counter. Binding results are expressed as percent of specific binding in comparison with the 100% of initial incubated activity and extrapolated to an infinite quantity of antigen as determined by Lindmo et al. [44].

### Direct cell death and complement-dependent cytotoxicity

Complement dependent cytotoxicity (CDC) and direct cell death were determined using the alamar blue vital dye (Biosource International, Camarillo, CA, USA) essentially as described [45]. Briefly, 20 000 cells/well were plated in 100 μL in quadruplicate in 96-well plates in the presence of 10 μg/mL of mAbs in the presence (CDC) or absence (direct cell death) of 20% pooled human serum from normal donors. After 24h of incubation at 37°C, the total volume was brought to 300 μL in medium containing 1/10 volume of alamar blue solution (Biosource International, Camarillo, CA, USA). Incubation was carried on 8-16 hours at 37°C and the plates were read in a fluorimeter (Genios, Tecan, Männedorf, Switzerland) with excitation at 530 nm and emission at 590 nm. Cell death was calculated as percentage decrease in live cells after subtracting background fluorescence given by medium alone.

### Inhibition of cell growth

10.000 JOK1-5.3 cells/well were plated in 96 well plates in presence or absence of 10 μg/ml antibodies. After 3 to 6 days at 37°C 5% CO2, 1/10 volume alamar blue solution was added and incubation continued for further 8 hours. Plates were read in a fluorimeter (Genios, Tecan) with excitation at 530 nm and emission at 590 nm. Inhibition of cell growth was calculated as percentage decrease in live cells relative to untreated controls, after subtracting background fluorescence.

Another test of the effects of mAbs on cell proliferation was made by measuring the incorporation of the radio-labelled thymidine analogue ^125^I-Iododeoxyuridine (^125^I-IdUrd). In brief, after the different times of incubation with individual Mabs or pair of mAbs at a final total concentration of 75 mg/ml of mAbs the target cells, JOK1-5.3 (2'500 cells per well of 96 wells culture plates) were further incubated for 4 hours at 37°C with 5kBq of ^125 ^I-IdUrd. After thorough washing, cell-pellet associated radioactivity was measured, using a γ-counter and cell proliferation inhibition was calculated on a titration curve established with known concentrations of untreated control cells. All tests were performed in triplicate and repeated at least 2 times.

### Phagocytosis

Phagocytosis was performed, as described previously, by differentiating monocytes to macrophages *in vitro *by 7 day culture in presence of rhM-CSF [27]. Briefly, CD14+ monocytes were plated n 8-well Lab Tek chamber slides (Cebio, Milan, Italy) at 200 000 cells/well and allowed to differentiate as above. B-CLL target cells were incubated in presence or absence of antibody and then added to macrophages at a 1:1 ratio in RPMI 1640/10% FBS. After 2 h at 37°C, cells were fixed and stained with Diff Quick and percentage phagocytosis was measured by counting macrophages having engulfed one or more B-CLL.

### ADCC

NK cells were positively selected from Ficoll-Isopaque purified PBMC using RosetteSep Human NK cell enrichment cocktail as recommended by the manufacturer. JOK1.5.3 or other target cells (JOK1 wild type, Jurkat or normal PBL) were first labelled for 10 min at 37°C in the dark, with CSFE (CellTrace CFSE Cell Proliferation Kit, C34554, Invitrogen, Paiseley, UK). Labelled target cells were aliquoted into a 96 well culture plate (25 000 per 50 μL) and appropriate dilution of chimeric antibodies or control human Ig_1 _was also added to the plate. Plates were incubated for 20min at 4°C. Next, NK cells were added to the wells at a 10:1 ratio. Plates were incubated for 4 hr at 37°C. After incubation each well was transferred into a 5ml tube and 7AAD was added to each tube (Invitrogen). After a final 15min incubation at room temperature in the dark, tubes were analysed by flow cytometry. Target cells were gated by their CFSE labelling, while the percentage of dead cells among target cells was identified by 7AAD labelling.

### *In vivo *studies

SCID CB-17 mice, aged 6-8 weeks, were purchased from the Charles River Breeding Laboratories (Wilmington, MA, USA mice used in the i.p. model), or Harlan (Boxmeer, Netherlands, mice in the i.v. model). Mice were kept under specific pathogen-free conditions in a separate facility using autoclaved cages of micro-isolator units and fed with irradiated solid food and sterilised water. All protocols were approved by the Institutional Ethics Committee for Animal Experimentation of Brittany (authorization b-2005-SL-04) and Swiss legislation (authorization VD 2013).

For the intraperitoneal model, 5 × 10^6 ^JOK1-5.3 cells in exponential growth were inoculated by 2 bilateral intra-peritoneal injections. Treatment consisted of 2 i.p. injections of 0.5mg of each mAb 24h and 92h after grafting. Controls mice were injected with PBS or an isotype matched irrelevant IgG_1_. For experiments involving the IgG_2a _anti-CD5 antibody, control mice grafted with JOK1-5.3 (CD5+) were injected with PBS, and, in a parallel experiment, other control mice were grafted with wild type JOK1 cells (CD5-) and tested with anti-CD5 antibody treatment and compared with PBS injection. Animals were monitored three times per week for tumour appearance, bodyweight measurement and signs of disease (ascites, cachexia). Subcutaneous tumour volume was measured with the formula (length × width × 2/3 width)/2. Experiments using combination treatments consisted of 2 injections i.p. of 0.25 mg of each mAb (0.5 mg total) 24 h and 96h after grafting. Combination treatments were compared to single agent treatment consisting of 2 injections of 0.5 mg each from the same mAb at 24 h and 96 h.

For the intravenous model, 10 × 10^6 ^JOK1-5.3 cells suspended in 0.1 ml PBS were injected i.v. into the tail vein. Mice were randomly divided into groups and injected i.v. with 0.25 mg of either irrelevant IgG_1 _or mAb on days 3, 5, 7, and 11. In combination experiments 0.25 mg of each mAb were coinjected i.v.. Mice were monitored daily for the presence of hind-leg paralysis and in that case sacrificed and scored as dead.

### Animal survival analysis

Kaplan-Meier plots and statistical analysis were performed using the GraphPad Prism 5 software and the log-rank (Mantel-Cox) test. Differences between curves were considered significant if p value was < 0.05, and highly significant if p < 0.005.

## Competing interests

The authors declare that they have no competing interests. The 3 mAbs described were protected by a patent taken by the Institut National de la Santé et de la Recherche Médicale (INSERM) Paris.

## Authors' contributions

PAA and SL performed the *in vivo *experiments using the i.p. and i.v. models, respectively. PAA performed the immunoreactivity evaluation of the mAbs and some cell proliferation/apoptosis assays. JG and LBologna performed extensive cell proliferation/apoptosis, CDC, and phagocytosis experiments. LB was responsible for the development and with MC for the initial characterisation of the 3 mAbs used in the study, and LB performed the ADCC experiments. JPM, JG, LB, PAA, FB and SL drafted the manuscript. FB and ABD were responsible for the supervision and training of PAA. DV participated in the *in vivo *and *in vitro *experiments performed in Lausanne. MK was responsible for the production of radiolabelled compounds. JK, CB, LB, MC and JPM participated in the initial conception, design and supervision of the study. All authors read and approved the final manuscript.
